# Reference Values of D-Dimers and Fibrinogen in the Course of Physiological Pregnancy: the Potential Impact of Selected Risk Factors—A Pilot Study

**DOI:** 10.1155/2020/3192350

**Published:** 2020-05-24

**Authors:** Aldona Siennicka, Magdalena Kłysz, Kornel Chełstowski, Aleksandra Tabaczniuk, Zuzanna Marcinowska, Paulina Tarnowska, Jolanta Kulesza, Andrzej Torbe, Maria Jastrzębska

**Affiliations:** ^1^Department of Laboratory Diagnostics, Pomeranian Medical University, Szczecin, Poland; ^2^Department of Obstetrics and Gynecology, Pomeranian Medical University, Szczecin, Poland

## Abstract

Pregnancy predisposes to thrombotic hemostasis, reflected in the laboratory as, e.g., increased levels of D-Dimers and fibrinogen, but in physiological pregnancy, the risk of venous thrombosis does not increase. Risk may increase if gestational diabetes mellitus (GDM) or nicotinism coexists. Study aims were to determine reference values for D-Dimers and fibrinogen concentrations in each trimester of pregnancy, corrected for GDM and nicotinism. *Subjects and Methods*. The study involved 71 pregnant women aged 25-44 y. Venous blood was collected three times: in the first (11-14 weeks), second (20-22 weeks), and third (30-31 weeks) trimesters. D-Dimer concentrations were determined by an enzyme-linked fluorescence assay, fibrinogen concentrations by a coagulation method according to Clauss. *Results*. Significant increases in D-Dimers and fibrinogen concentrations were observed, increasing with successive trimesters (*p* ANOVA < 0.0001). Furthermore, a positive correlation between D-Dimers and fibrinogen was detected in the second trimester of pregnancy (*r* = 0.475; *p* < 0.0001). In addition, a significantly higher fibrinogen concentration was found in women with GDM compared to without GDM (*p* = 0.0449). Reference ranges for D-Dimers were established, in trimester order, as follows: 167-721 ng/mL, 298-1653 ng/mL, and 483-2256 ng/mL. After adjusting for risk factors, significantly higher D-Dimer values (mainly second and third trimesters) were obtained: 165-638 ng/mL, 282-3474 ng/mL, and 483-4486 ng/mL, respectively. Reference ranges for fibrinogen were, in trimester order, 2.60-6.56 g/L, 3.40-8.53 g/L, and 3.63-9.14 g/L and, after adjustment for risk factors, 3.34-6.73 g/L, 3.40-8.84 g/L, and 3.12-9.91 g/L. *Conclusions*. We conclude that the increase in D-Dimers and fibrinogen levels in women with physiological pregnancy was compounded by gestational diabetes (GDM) and nicotinism. Therefore, D-Dimers and fibrinogen pregnancy reference values require correction for these risk factors.

## 1. Introduction

A normal pregnancy is characterized by changes in blood coagulation and fibrinolysis with a thrombotic nature, often referred to as physiological hypercoagulability. The results of many studies have shown that increased thrombotic activity during pregnancy is characterized by significant hyperfibrinogenemia, an increase in the activity of plasma coagulation factors, mainly VII, VIII, IX, X, and XII; a decrease in the concentration of the natural clotting inhibitor protein S; and by intensification of the processes of adhesion and platelet aggregation [1, 2].

The consequence of high procoagulation activity is increased fibrin turnover, as indicated by increasing concentrations of D-Dimers (D-D), recognized as the most sensitive markers of secondary fibrinolytic activation, with successive trimester [3]. Changes in hemostatic systems occur gradually in normal pregnancy, reaching the highest degree of hypercoagulability in the third trimester and disappearing slowly during puerperium [2]. They should be considered an adaptive mechanism protecting a pregnant woman against delivery hemorrhage and generally do not have any clinical implications. In one recent meta-analysis, the most common complication, venous thromboembolism (VTE), was found to have low incidence, estimated at 1.2 per 1000 births [4]. However, the risk of pulmonary embolism (which often results from VTE) has been found to be higher and occurs 4-6 times more often than in nonpregnant women of similar age [5].

An increase in thrombotic risk occurs most often in pregnant women with cardiovascular risk factors (nicotinism, diabetes, hypertension, and overweight/obesity), with prolonged immobilisation, use of hormonal oral contraception before pregnancy, and/or with pathological pregnancy factors (premature placental abruption, preeclampsia) [6]. Nicotinism and diabetes, in combination with hyperestrogenism, are particularly strong risk factors for VTE [7]. Similarly, an increased risk of VTE during pregnancy is observed in women with concomitant carbohydrate disorders [8]. Venous thromboembolism is manifested most often as deep vein thrombosis (DVT) of the lower limbs, or as pulmonary embolism (PE), or both together. Pulmonary embolism remains the main cause of perinatal mortality in developed countries and accounts for around 10-20% of pregnancy-related deaths [9]. The occurrence of VTE is associated with a significant risk of serious complications in pregnant women, such as massive hemorrhage or postthrombotic syndrome.

The diagnosis of VTE in pregnant women is particularly difficult due to nonspecific clinical symptoms (limb edema, shortness of breath) as well as the lack of standard diagnostic procedures for the exclusion of VTE based on low D-Dimer levels. The assessment of thrombotic risk during pregnancy by determining D-Dimers and fibrinogen concentrations is of limited value at present using ranges from the general population. This is due, as mentioned earlier, to the physiological and gradual increase in both D-Dimers and fibrinogen (Fb) that is observed in pregnant women. For this reason, during pregnancy, it is not possible to use the reference ranges of D-Dimers and fibrinogen concentrations determined for the general population [10]. Therefore, the main goal of our research was to determine reference values for D-Dimers and fibrinogen concentrations in the three trimesters of physiological pregnancy. Due to the presence of women with risk factors for hypercoagulability (smoking, gestational diabetes) that could potentially affect D-Dimers and fibrinogen concentrations, an additional objective was to correct for these risk factors.

## 2. Materials and Methods

### 2.1. Subjects

The study involved 71 pregnant women between the ages of 25 and 44 under the care of the Prenatal Research Outpatient Clinic of Obstetrics and Gynecology at the Pomeranian Medical University (PMU) in Szczecin, Poland. Of the 71 pregnant women who decided to participate in the study in the first trimester, 67 subjects participated in the second trimester and 62 in the third trimester. Questionnaires and interviews were completed regarding carbohydrate disorders, hypertension, nicotinism, and aggravating family history of venous thromboembolism in first- and/or second-degree relatives. The questionnaires also obtained information for calculation of the body mass index. The exact characteristics of pregnant women are given in Table 1. Reference ranges were calculated for the entire study group (Group A), as well as for the following overlapping subgroups: nonsmoking and nondiabetic (Group B), nonsmoking with or without diabetes (Group C), and with smoking and diabetes (Group D). Interactions between diabetes and trimester, and between smoking and trimester, were also determined by dividing the entire study group into ±diabetes or ±smoking.

The study was approved by the Bioethics Committee at PMU. Condition for participation in the study was written consent. The exclusion criteria are history of venous thrombosis, heart defects or hypertension, autoimmune or cancerous diseases, kidney or liver diseases, hematological diseases, acute and chronic infections, long-term immobilisation, and anticoagulants or hormonal contraceptives or pathological pregnancy (preeclampsia, premature placental abruption).

### 2.2. Blood Collection and Laboratory Analyses

Venous blood was collected three times: in the first (11-14 weeks), second (20-22 weeks), and third (30-31 weeks) trimesters, into two tubes (S-Monovette, Sarstedt), which differed in anticoagulant. One tube contained 5 mL blood and 3.2% sodium citrate in a 9 : 1 ratio, which was centrifuged (15 min, 1500 × g) to obtain platelet-poor plasma, for determination of D-Dimers (D-D) and fibrinogen (Fb) concentrations and activated partial thromboplastin times (APTT). The second tube contained 2 mL blood and sodium edetate (EDTA) for blood morphology. D-Dimer concentrations were determined using an enzyme-linked fluorescence assay (ELFA; D-Dimer Exclusion II (DEX2) kit and VIDAS analyzer; bioMerieux, France). Fibrinogen concentrations and APTT were measured using ready-made reagent kits (with an ACL ELITE analyser), for Fb (Q.F.A. Thrombin) and for APTT (APTT-P-REAGENT kit; Instrumentation Laboratory, Germany). Blood morphology was assessed (using an ABX Micros 60 hematology analyser; Horiba). APTT and blood morphology determinations were made with plasma/blood samples within 2 hours of blood collection, while D-Dimer and fibrinogen concentrations were measured in previously frozen (-30°C) plasma samples.

### 2.3. Statistical Analyses

Data were statistically analysed using commercial software (Statistica v.12.0; StatSoft, Tulsa, Oklahoma, USA). Kolmogorov-Smirnov tests were used to test for normality, and logarithmic transformations applied when necessary (for D-Dimer and fibrinogen levels). For statistical calculations, analysis of variance was used for repeated measures, either one-factor ANOVA for assessment of the impact of pregnancy on the concentrations of D-Dimers and fibrinogen or two-factor ANOVA for assessment of interactions between diabetes and the course of pregnancy as well as smoking and pregnancy. Additionally, least significant difference tests were used post hoc. Linear correlations were assessed using Pearson's correlation coefficient. In order to establish reference values for D-Dimer and fibrinogen concentrations, the percentile method was adopted and normal values were assumed to lie between the 2.5 and 97.5 percentile. A *p* value of <0.05 was considered significant. Due to the small size of some groups, values with 0.05 < *p* < 0.1 as indicative of possible trends were also considered.

## 3. Results

Characteristic, hemostatic, and hematology parameters of the pregnant women are shown (Tables 1 and 2). The concentrations of D-Dimers (D-D) and fibrinogen (Fb) increased with successive trimesters (Table 2) and characteristic of physiological pregnancy and accompanied by normal APTT clotting times and hematological parameters.

**Figure 1:**
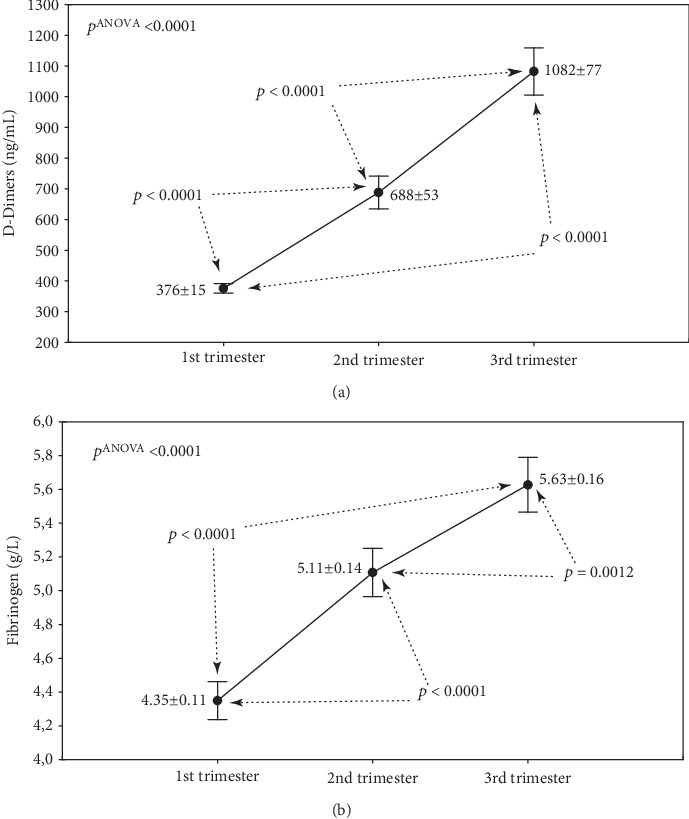
The effect of physiological pregnancy on the concentrations of D-Dimers (a) and fibrinogen (b). Abbreviations: *p* ANOVA: *p* values from one-factor ANOVA; 1st trimester (*n* = 71), 2nd trimester (*n* = 67), and 3rd trimester (*n* = 62).

**Figure 2:**
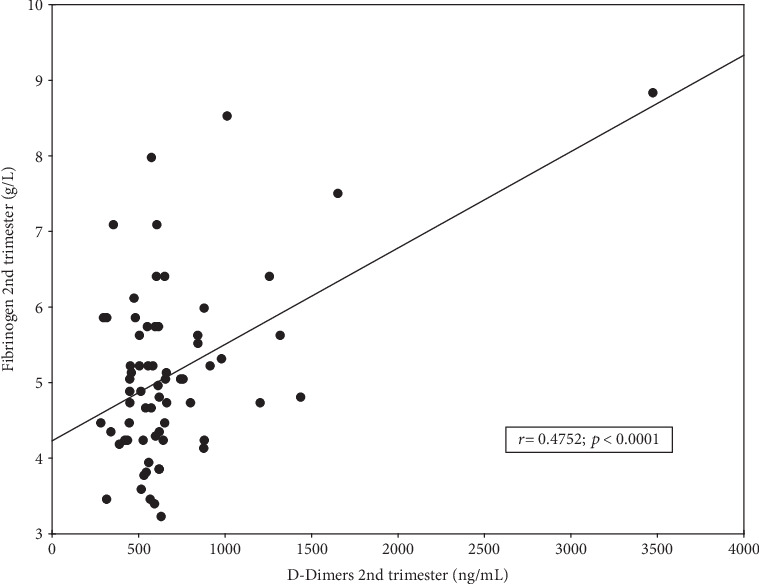
Correlation between the concentrations of D-Dimers and fibrinogen in the second trimester (*n* = 67) of physiological pregnancy.

There were highly statistically significant differences between different trimesters for D-Dimers and also fibrinogen concentrations (*p* ANOVA < 0.0001; Figures 1(a), 1(b)). Post hoc tests showed significantly higher concentrations in the 2nd trimester compared to the first and significantly higher in the 3rd compared to the first or second (all differences with *p* < 0.0001 except for Fb: 2nd vs. 3rd trimester: *p* = 0.0012). There was a strong positive correlation between concentrations of D-Dimers and fibrinogen (Figure 2), observed mainly in the 2nd trimester (*r* = 0.4752; *p* < 0.0001).

**Figure 3:**
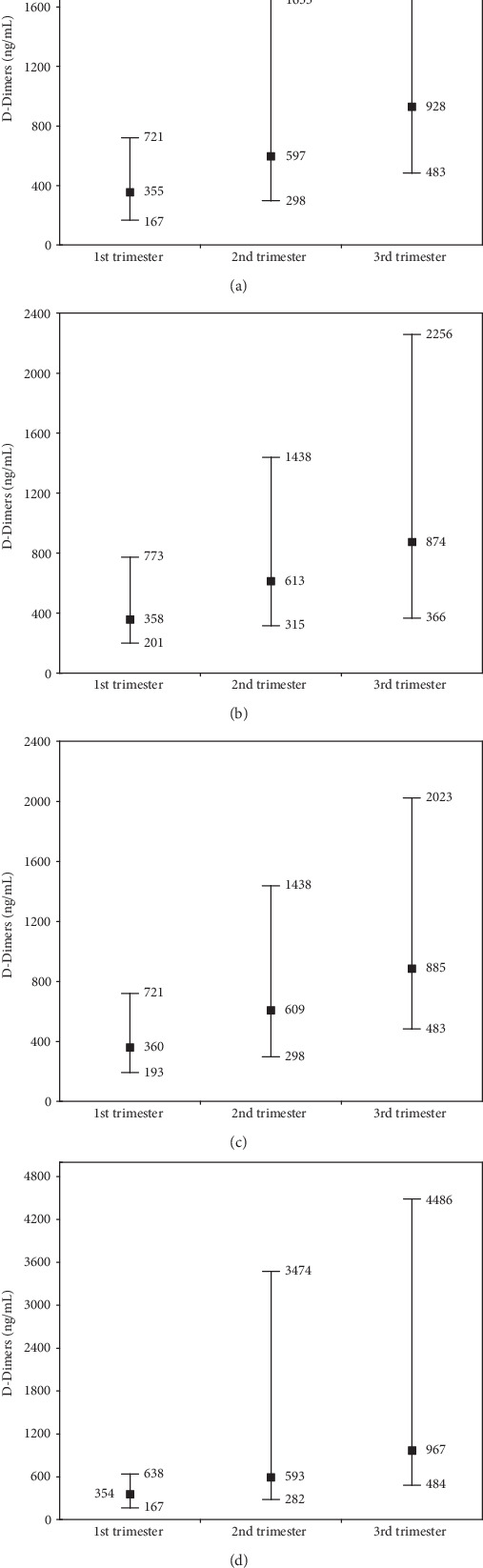
The range of reference values for the concentrations of D-Dimers in individual trimesters of physiological pregnancy. Values determined in four groups (a–d) depending on the coexistence of risk factors (smoking and gestational diabetes mellitus; GDM). Abbreviations: Group A: all women (1st trimester *n* = 71, 2nd trimester *n* = 67, and 3rd trimester *n* = 62); Group B: nonsmoking and nondiabetic GDM^−^ (1st trimester *n* = 40, 2nd trimester *n* = 37, and 3rd trimester *n* = 35); Group C: nonsmoking and nondiabetic/diabetic GDM^−^/GDM^+^ (1st trimester *n* = 51, 2nd trimester *n* = 48, and 3rd trimester *n* = 45); Group D: smoking and nondiabetic/diabetic GDM^−^/GDM^+^ plus nonsmoking and diabetic GDM^+^ (1st trimester *n* = 32, 2nd trimester *n* = 31, and 3rd trimester *n* = 28).

**Figure 4:**
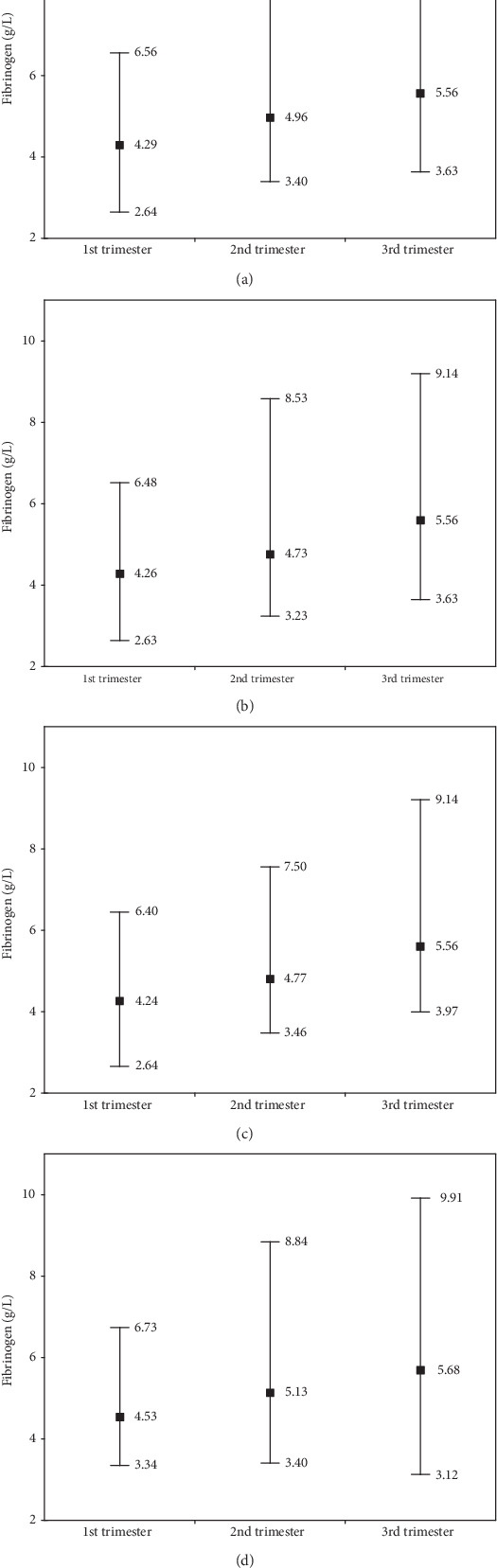
The range of reference values for the concentration of fibrinogen in individual trimesters of physiological pregnancy. Values determined in four groups (a–d) depending on the coexistence of risk factors (smoking and gestational diabetes mellitus; GDM). For Abbreviations, group definitions, and sample sizes, see Figure 3 legend.

Reference value ranges for D-Dimer and fibrinogen concentrations for the entire study group over all trimesters, without adjusting for smoking and gestational diabetes, are shown in Figures 3(a) and 4(a) and after adjustment in Figures 3(b)–3(d) and 4(b)–4(d).

Reference values for D-D without adjustment for the entire group (Figure 3(a)) for successive trimesters were 167-721, 298-1653, and 483-2256 ng/mL. Similar reference ranges were obtained for women without risk factors (Figure 3(b)) or with diabetes as one risk factor (Figure 3(c)). However, attention is drawn to those with two risk factors (Figure 3(d)) which, after adjustment, had considerably higher D-D reference values (especially after the 1st trimester): 165-638, 282-3474, and 483-4486 ng/mL, respectively. Similar trends were also found for Fb reference ranges (Figure 4). For the entire study group (Figure 4(a)), reference value ranges in successive trimesters were 2.64-6.56, 3.40-8.53, and 3.63-9.14 g/L. Similar ranges were obtained in Groups B and C (Figures 4(b) and 4(c)). As with the D-D values, higher Fb concentrations, after correction, were found in Group D: 3.34-6.73, 3.40-8.84, and 3.12-9.91 g/L.

**Figure 5:**
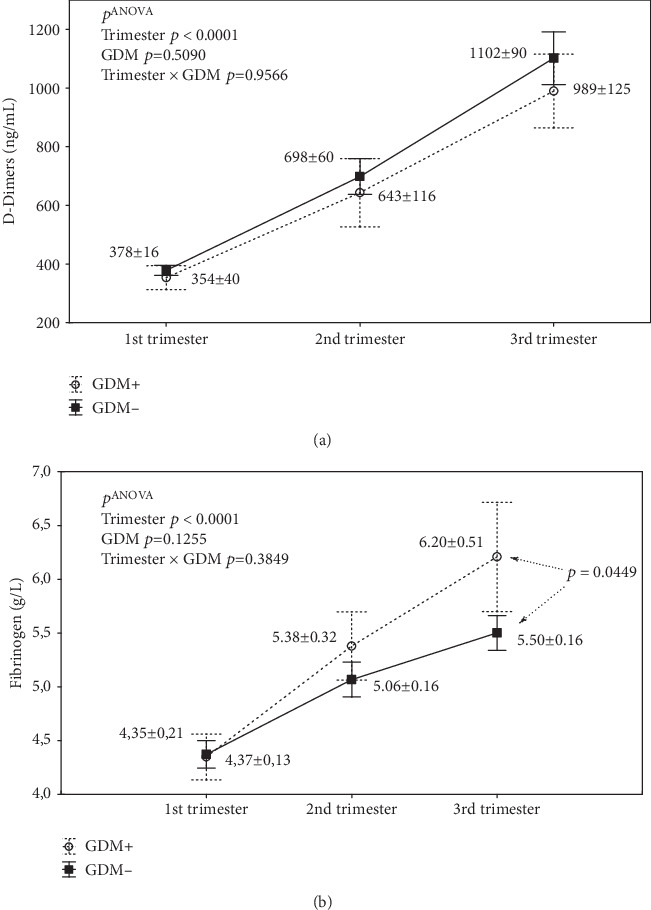
Analysis of the interaction of gestational diabetes mellitus (GDM) and the course of pregnancy (trimesters) on the concentrations of D-Dimers (a) and fibrinogen (b). Abbreviations: *p* ANOVA, *p* values from two-factor ANOVA. Statistical analysis between GDM^+^/GDM^−^ for fibrinogen (b), *p* values from post hoc least significant difference (LSD) tests. No significant differences in the LSD test for D-Dimers (a). Diabetic group GDM^+^ (1st trimester *n* = 12, 2nd trimester *n* = 12, and 3rd trimester *n* = 11). Non-diabetic group GDM^−^ (1st trimester *n* = 59, 2nd trimester *n* = 55, and 3rd trimester *n* = 51).

**Figure 6:**
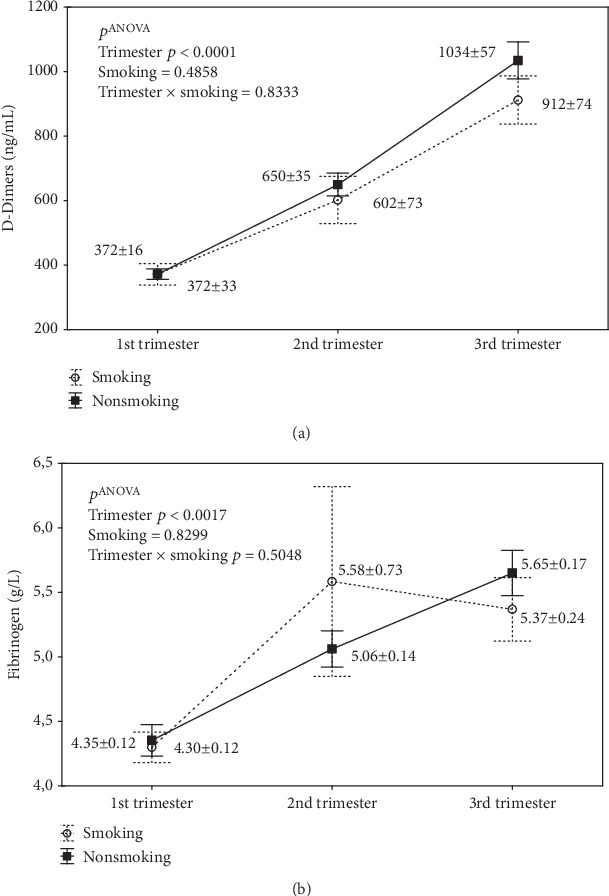
Analysis of the interaction of smoking and the course of pregnancy (trimesters) on the concentrations of D-Dimers (a) and fibrinogen (b). Abbreviations: *p* ANOVA, *p* values from two-factor ANOVA. No significant differences in the LSD tests (a, b). Smoking group (1st trimester *n* = 6, 2nd trimester *n* = 5, and 3rd trimester *n* = 5). Nonsmoking group (1st trimester *n* = 65, 2nd trimester *n* = 61, and 3rd trimester *n* = 57).

With two-factor ANOVA (Figures 5 and 6) including the interaction of risk factors (GDM or smoking) and trimester, no interaction was found between gestational diabetes (GDM) and trimester for both D-D (*p* ANOVA = 0.9566; Figure 5(a)) and for Fb (*p* ANOVA = 0.3849; Figure 5(b)). However, there was a close relationship between D-D and Fb increase with trimester for all groupings (*p* ANOVA < 0.0001).

These results are consistent with the results shown in Figure 1. However, attention should be drawn to the effect of GDM on Fb concentration (Figure 5(b)). Although the relationship between GDM and the increase in Fb concentration was not significant by ANOVA (*p* ANOVA = 0.1255), post hoc tests showed significantly higher Fb concentrations found in those with GDM compared to without (*p* = 0.0449).

Two-way ANOVA showed no interaction between smoking and trimester (Figure 6), and post hoc tests showed no significant differences in D-D and Fb levels between smokers and nonsmokers. A close relationship between D-D and Fb increase with successive trimesters was demonstrated, for D-D, *p* ANOVA < 0.0001 (Figure 6(a)) and, for Fb, *p* ANOVA = 0.0017 (Figure 6(b)).

## 4. Discussion

Pregnancy is associated with numerous physiological changes that affect the functioning of almost every organ system, including changes in hemostasis [11]. In the case of hemostasis, these changes are significant enough to significantly affect the results of laboratory tests. It is often emphasized in the medical literature that D-Dimer concentrations, which are products of the action of plasmin on stabilized fibrin, and fibrinogen (Fb), are increased in pregnant women compared to nonpregnant women, and their increase progresses with the stage of pregnancy [12–14].

The results of this study confirm these reports. As expected, the concentrations of D-D and Fb in the pregnant women progressively, and statistically significantly, increased and reached the highest values in the third trimester. It is worth emphasizing that elevated levels of D-Dimer may appear both in normal pregnancy and in pregnancy complicated by, e.g., hypertension or gestational diabetes. From this, the possibility of using D-Dimer reference ranges determined for the general population (men and nonpregnant women) for the diagnosis of venous thrombotic disease during pregnancy is limited [15]. Tests for the determination of D-Dimers are characterized by a high diagnostic sensitivity and high negative predictive value, estimated at 93-96%, but a much lower diagnostic specificity (38-42%), and therefore, for the diagnosis of venous thrombotic disease these mainly allow exclusion [16, 17]. It should also be remembered that for the exclusion of venous thromboembolism based on D-Dimer concentration tests, the most important diagnostic value of the test is the cut-off value, which is a compromise between sensitivity and specificity.

With nonpregnant women, exclusion of deep vein thrombosis (DVT) or pulmonary embolism (PE) based on low D-D concentration most commonly uses a cut-off value of <500 ng/mL. With pregnant women, cut-off values should be determined for each trimester, and according to some reports, possible cut-off values could be of the order of 286, 457, and 644 ng/mL, respectively [18]. It should be emphasized that in the present study, 75% of women in the second trimester and 97% of women in the third trimester exceeded these cut-offs significantly, which in the absence of clinical symptoms of venous thrombosis indicates a lack of D-D significance, using these cut-offs, in the diagnosis of venous thrombosis during pregnancy. In our study, it was also observed that an increased D-D concentration across the entire sample studied had a significant positive correlation with fibrinogen concentration increase, especially in the 2nd trimester. This correlation is found in the literature concerning all three trimesters [19]. There are also previous studies in which no such significant correlation was found [3, 10, 20]. In view of these contradicting results, it is possible to view other relationships between these parameters. For example, it has been shown that the ratio between D-Dimers and fibrinogen could be a useful indicator in the diagnosis of pulmonary embolism, which remains the main cause of death in pregnant women [21].

As mentioned above, the reference values of the tested parameters (D-D and Fb) obtained in the present study were significantly increased in all trimesters and differed from those established for adult nonpregnant women [22]. In the case of D-Dimers, the concentrations for the entire group of pregnant women fell within the following ranges: first trimester 167-721 ng/mL, second trimester 298-1653 ng/mL, and third trimester 483-2256 ng/mL. These results were similar to those published in the literature [3, 23, 24]. But there are also studies that do not confirm the reference values of D-Dimers obtained in our own research [25]. It seems that differences in the reference values of D-Dimers in pregnant women may be due to, e.g., different pregnancy risk factors in the samples studied.

The present study established the following ranges for Fb concentrations which could be proposed as reference ranges: first trimester 2.64-6.56 g/L, second trimester 3.40-8.53 g/L, and third trimester 3.63-9.14 g/L. Such high Fb concentrations, especially the upper reference value, are confirmed only in some literature [19]. In other studies, the concentration ranges were lower [26–28]. These differences may result from different ethnicities of sampled populations. However, it is also worth mentioning that some researchers have obtained even higher concentration ranges of Fb compared to the present study [3]. This could be due to different methods for determining Fb concentrations or to later sampling points within the same trimesters.

Analysis of coexisting risk factors (smoking and gestational diabetes) may also be useful in explaining differences between studies, which was an additional goal of the present study. As a reminder, in addition to the entire sample (1st group), three overlapping groups of pregnant women were also analysed: women without risk factors (2nd group), women with one risk factor (3rd group), and women with two risk factors (group 4). The reference values were adjusted for risk factors, as shown in Figures 3 and 4. The group with two risk factors (group 4) is of note. These had the highest concentrations of D-Dimers and fibrinogen in the 2nd and 3rd trimesters. In our opinion, this may have been due to the simultaneous effects of gestational diabetes (GDM) and smoking. According to the available literature, gestational diabetes may cause blood clotting from increased platelet activation, increased coagulation factor synthesis, including fibrinogen, and reduced fibrinolytic activity [29]. The occurrence of hyperfibrinogenemia increased, as in the literature, with successive trimester, and this was particularly marked with gestational diabetes [29]. In previous studies, a higher concentration of fibrinogen has been also observed with GDM compared to without GDM [30]. However, previous studies have not shown clear changes in levels of proinflammatory cytokines (IL-2, IL-6, and IL-8), which might indicate a different cause of pregnancy hyperfibrinogenemia complicated by GDM than inflammation [30].

As mentioned earlier, the present study showed a tendency for higher fibrinogen concentrations with GDM compared to without GDM, most strongly expressed in the 3rd trimester. In our opinion, this relationship confirms the hypothesis that gestational diabetes is an important risk factor for hyperfibrinogenemia and the thrombotic state, which increases with the course of pregnancy.

According to literature reports, smoking is an important risk factor for a thrombotic state in pregnancy, in addition to gestational diabetes [7, 8]. Some studies have shown that smoking contributes to the formation of systemic inflammatory response, as indicated by elevated levels of acute-phase proteinss (mainly C-reactive protein and fibrinogen) and an increase in absolute leukocyte counts [31–34]. These reports have suggested nicotinism-induced inflammation as an important factor in the progression of the thrombotic state, as indicated by elevated D-Dimer levels among smokers [33]. Previous studies have shown that women, compared to men, might be more sensitive to components of tobacco smoke [35]. Smoking women, compared to nonsmoking women and men, have increased platelet turnover resulting from increased platelet aggregation, confirming the effect of smoking on progression of the thrombotic state [35]. However, with the analysis of the impact of smoking in our study, no significant interaction between cigarette smoking and pregnancy was demonstrated for both D-Dimer and fibrinogen levels. Despite this, attention should be paid to the significant impact of nicotinism with gestational diabetes (group 4), with significantly higher concentrations for both D-Dimers and fibrinogen.

### 4.1. Limitation of the Study

One limitation of this study is the relatively small sample sizes. Since no reports have been found in the available literature regarding the correction of reference values of D-Dimers and fibrinogen with the risk factors of GDM and smoking, we believe that despite the small number of women in the study groups, the values should be taken into account. Undoubtedly, a strength of the study is that the assessment of D-Dimer and fibrinogen levels in all three trimesters was performed in the same women, eliminating the impact of interindividual variability on the results obtained.

## 5. Conclusions

In conclusion, it was found that physiological pregnancy is accompanied by progressive increases in D-Dimer and fibrinogen levels and this increase appeared to be compounded by risk factors for thrombosis, in this case by gestational diabetes and nicotinism. Therefore, D-Dimer and fibrinogen reference values should be corrected for these risk factors. In addition, we conclude that pregnant women with gestational diabetes may have an increased predisposition to the development of perinatal venous thrombosis due to more pronounced hyperfibrinogenemia.

For pregnant women, the following D-Dimer reference value ranges are proposed: in the first trimester of pregnancy, 167-721 ng/mL; in the second trimester of pregnancy, 298-1653 ng/mL; and in the third trimester of pregnanc,: 483-2256 ng/mL, and fibrinogen reference value ranges: in the first trimester, 2.64-6.56 g/L; in the second trimester, 3.40-8.53 g/L; and in the third trimester, 3.63-9.14 g/L. In those with diabetes and nicotinism, the reference ranges were higher, mainly in the 2nd and 3rd trimesters, as follows: D-Dimer ranges: 167-638 ng/mL (1st trimester), 282-3474 ng/mL (2nd trimester), and 484-4486 ng/mL (3rd trimester), and fibrinogen ranges: 3.34-6.73 g/L (1st trimester), 3.40-8.84 g/L (2nd trimester), and 3.12-9.91 g/L (3rd trimester).

## Figures and Tables

**Table 1:** Base demographics and clinical characteristics of the study group of pregnant women.

	Pregnant women *N* = 71
Age (years, mean ± SD)	34.4 ± 4.5
BMI (kg/m^2^) (mean ± SD)	24.4 ± 4.3
Smoking before pregnancy (*N*/%)	20/28
Smoking during pregnancy (*N*/%)	6/8
IFG (*N*/%)	10/14
NIDDM (*N*/%)	1/1
GDM (*N*/%)	12/17
Hypertension (*N*/%)	0/0
Family history of VTE (*N*/%)	5/7

Abbreviations: IFG: impaired fasting glucose; NIDDM: noninsulin-dependent diabetes mellitus; GDM: gestational diabetes mellitus; VTE: venous thromboembolism.

**Table 2:** Descriptive statistics of laboratory parameters determined in the study group of women, divided into trimesters.

Parameter	First trimester *N* = 71	Second trimester *N* = 67	Third trimester *N* = 62
	Mean ± SD	Mean ± SD	Mean ± SD
D-D (ng/mL)	376 ± 129	688 ± 436	1082 ± 606
Fb (g/L)	4.35 ± 0.95	5.11 ± 1.17	5.63 ± 1.28
APTT (s)	33.3 ± 3.2	33.2 ± 3.1	33.0 ± 3.2
PLT (G/L)	208 ± 45	213 ± 75	220 ± 66
WBC (G/L)	7.7 ± 1.7	8.5 ± 2.0	9.2 ± 2.4
RBC (T/L)	3.81 ± 0.32	3.62 ± 0.39	3.83 ± 0.45
HGB (mmol/L)	6.9 ± 0.6	6.6 ± 0.8	7.0 ± 1.2
HCT (L/L)	0.32 ± 0.03	0.32 ± 0.04	0.34 ± 0.05

Abbreviations: D-D: D-Dimers; Fb: fibrinogen; APTT: activated partial thromboplastin time; PLT: platelet count; WBC: white blood cell count; RBC: red blood cell count; HGB: hemoglobin; HCT: hematocrit.

## Data Availability

The data used to support the findings of this study are available from the corresponding author upon request.
